# The gray zone of thyroid nodules: Using a nomogram to provide malignancy risk assessment and guide patient management

**DOI:** 10.1002/cam4.3866

**Published:** 2021-03-24

**Authors:** Elham Yousefi, Gloria H. Sura, Jonathan Somma

**Affiliations:** ^1^ Department of Pathology SUNY Downstate Medical Center Brooklyn NY USA; ^2^ Department of Pathology LSU Health Sciences Center New Orleans LA USA

**Keywords:** fine‐needle biopsy, risk assessment, thyroid neoplasms, thyroid nodules, thyroidectomy, ultrasonography

## Abstract

**Background:**

Thyroid nodules have a low prevalence of malignancy and most proven cancers do not behave aggressively. Thus, risk‐stratification of nodules is a critical step to avoid surgical overtreatment. We hypothesized that a risk management system superior to those currently in use could be created to reduce the number of clinically indeterminate nodules (i.e., the “gray zone”) by concurrently considering the malignancy risks conferred by clinical, ultrasonographic, and cytologic variables.

**Methods:**

Thyroidectomy cases were reviewed from three institutions. Their benign versus malignant outcome was used to evaluate the variables for correlation. A binary logistic regression model was trained and, using indeterminate nodules with Bethesda III and IV results, validated. A scoring nomogram was designed to demonstrate the application of the model in clinical practice.

**Results:**

One hundred thirty thyroidectomies (28% malignant) met inclusion criteria. The final logistic regression model included difficulty in swallowing, hypothyroidism, echogenicity, hypervascularity, margins, calcification, and cytology diagnosis as input parameters. The model was highly successful in determining the outcome (*p* value: 0.001) with a R^2^(Nagelkerke) score of 0.93. The area under the curve as determined by receiver operating characteristics was 0.91. The accuracy of the model on the training dataset was 93% (sensitivity and specificity 92% and 96%, respectively) and, on the validation dataset, 80% (sensitivity and specificity 91% and 67%, respectively).

**Conclusions:**

We report a model for risk assessment of thyroid nodules that has the potential to significantly reduce indeterminates and surgical overtreatment. We illustrate its application via a straightforward nomogram, which integrates clinical, ultrasonographic, and cytologic data, and can be used to create clear, evidence‐based management plans for patients.

## Graphical abstract

A nomogram based on clinical, radiological, and cytological variables provides individualized malignancy risk assessments and management plans for patients with thyroid nodules. This approach may translate into a reduction of indeterminate cytology results and decrease unnecessary surgical intervention.

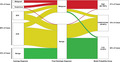

## 1 INTRODUCTION

In the United States, an estimated 18 million asymptomatic individuals have at least one thyroid nodule, and, in recent years, these have been increasingly incidental findings on imaging studies.^1^ While many factors play a role in defining cancer risk, precise risk‐stratification of thyroid nodules is a critical step for patient management because the prevalence of malignancy among nodules is at most 15% and because the majority of thyroid cancers do not behave aggressively.^2^

Some existing risk‐stratification systems, such as those advocated by the American Thyroid Association (ATA) and by the American College of Radiology (i.e., TI‐RADS), are intended to standardize ultrasonographic reporting and scoring of thyroid nodules. These generally serve to triage patients for thyroid fine‐needle aspiration (FNA) biopsy, which, in turn, assists in limiting surgical overtreatment.^3^, ^4^ While these systems can greatly simplify the interpretation of diagnostic sonography reports, there is not yet one universally accepted scoring system.^5^ Implemented about 10 years ago, “The Bethesda System for Reporting Thyroid Cytopathology” appears to be a more widely and consistently utilized risk‐stratification system, albeit with a different purpose.^6^ It arose from an international effort involving specialists from pathology, radiology, and endocrinology and represented a giant leap forward in the standardization of the preoperative diagnosis, reporting, and management of thyroid nodules. However, a “gray zone” still exists with approximately 20 percent of biopsied nodules remaining indeterminate after FNA, those falling in the Bethesda III and IV categories of “Atypia of Undetermined Significance” (AUS) and “Suspicious for Follicular Neoplasm” (SFN), respectively, and, while molecular testing is playing a role in informing management for these nodules, it is not without its difficulties including accessibility, expense, and imprecision.^7^, ^8^, ^9^

We hypothesized that a risk management system superior to The Bethesda System could be created by concurrently considering the malignancy risks conferred by clinical data, sonography, and Bethesda result, which might be of particular use in guiding management for gray zone nodules.

## 2 MATERIALS AND METHODS

In this retrospective cohort study, thyroidectomy cases accessioned within a 5‐year period starting January 1, 2014, were evaluated from three institutions: 1. University Hospital of Brooklyn (Brooklyn, New York), 2. Kings County Hospital Center (Brooklyn, New York), and 3. University Medical Center New Orleans (New Orleans, Louisiana). The inclusion criteria required that, within the year prior to each surgery, diagnostic thyroid ultrasonography data were available in the medical record, FNA biopsy of the thyroid had occurred in the same institution, and, where applicable, the FNA matched the laterality of the thyroidectomy. All FNA results were reported using The Bethesda System, and thyroidectomies with non‐diagnostic prior FNA results were excluded. This study was deemed as “exempt human subjects research” by the Institutional Review Boards.

For each case, numerous clinical and ultrasonographic variables were evaluated. Among the sonographic variables were nodule composition (e.g., spongiform, cystic, solid, mixed/complex), margin (e.g., smooth, irregular/lobulated, extra‐thyroid extension), and echogenic foci (e.g., comet‐tail artifact, macrocalcification, rim calcification, punctate foci/microcalcification). Variables, like nodule orientation (e.g., taller than wide), that were not commented on in an adequate number of cases were ultimately excluded and are not included in the tabulated data.

The histologic (i.e., thyroidectomy) diagnoses were reviewed and correlated with the prior FNA and ultrasound reports. All cases were categorized as either benign or malignant. Noninvasive follicular thyroid neoplasm with papillary‐like nuclear features (NIFTP) was considered a benign outcome as this low‐risk neoplasm is not expected to recur or metastasize after diagnostic lobectomy.^10^, ^11^ Histologic outcome was used to evaluate clinical, ultrasonographic, and cytologic variables for univariate correlation. Subsequently, using the package “rms” in R (version 3.6), a binary logistic regression model with a penalized maximum likelihood estimation was trained using the same variables from randomly drawn cases. The randomization was performed with stratification to ensure that adequate indeterminate cases (AUS and SFN cases) would remain for a validation dataset. A backward stepwise variable selection with Akaike information criterion (AIC) penalization was used to train the model and the binary outcome (benign versus malignant) was used as the response variable. The training model was internally validated using a bootstrap approach before the validation set was evaluated using the model. Based on the B‐coefficients of the significant parameters in the binary logistic regression, a scoring nomogram was designed to demonstrate the clinical application of the model using the nomogram function of the “rms” package.

## 3 RESULTS

**FIGURE 1:**
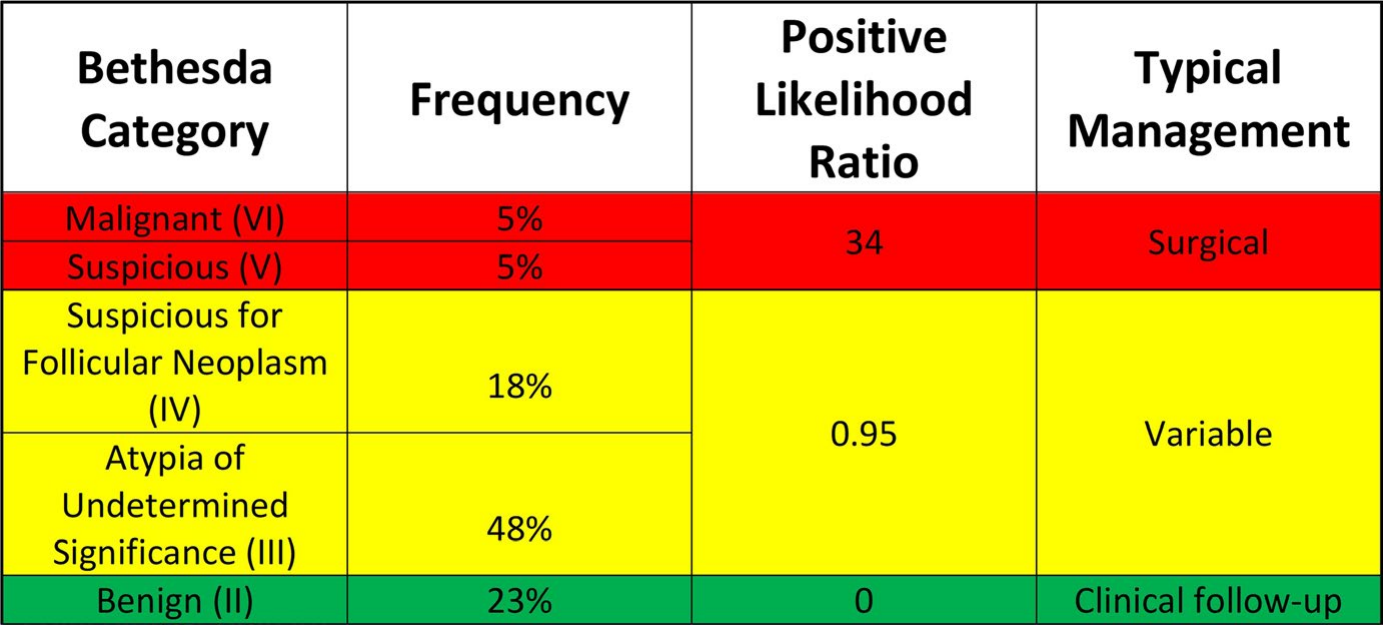
Bethesda fine‐needle aspiration (FNA) result distribution, positive likelihood ratio, and typical management (based on FNA result alone) of the 130 thyroidectomy cases. Indeterminates are highlighted in yellow

Of a total of 430 partial or total thyroidectomy cases evaluated, 130 met the inclusion criteria. The most common FNA result was AUS (62 cases) (Figure 1) and all AUS cases described thyroid follicular lesions rather than, for example, atypical lymphoid cells. Ninety‐four cases (72%) showed benign histology with the most common diagnosis being nodular hyperplasia (49 cases), followed by follicular adenoma (19 cases). Two had a diagnosis of NIFTP. Six showed only papillary microcarcinomas and these were all included in the benign group after correlation verified that these were incidental findings. Thirty‐six cases were malignant on thyroidectomy. Papillary carcinomas were most frequent (31 cases including one cribriform‐morular variant), followed by three follicular and two medullary carcinomas.

In the cases with benign histology, the most common cytology diagnosis was AUS, followed by benign (Table 1), and all of the benign FNAs were histologically benign including one of the NIFTPs. The most common cytology preceding a malignant outcome was again AUS and all malignant FNAs were confirmed. Only one suspicious FNA had a benign outcome (the other NIFTP).

**TABLE 1:** Univariate analysis of thyroid FNA results

Bethesda Category	Final Diagnosis: Benign	Final Diagnosis: Malignant	*p* Value
Benign	30 (32%)	0 (0%)	0.05
AUS	48 (51%)	14 (39%)	0.51
SFN	15 (16%)	9 (25%)	0.23
Suspicious for Malignancy	1 (1%)	6 (17%)	0.02
Malignant	0 (0%)	7 (19%)	0.02

Table 2 reports the univariate analysis of clinical variables; only difficulty swallowing and hypothyroidism were significant, and both were associated with a benign outcome. Of note, while 11 patients had presented with hypothyroidism, only 9 had use of thyroid replacement medication clearly recorded in their medical record. Forty‐three nodules had had a previous biopsy. These prior FNAs were diagnosed as follows: 5 unsatisfactory, 24 benign, 13 indeterminate (8 AUS and 5 SFN), and 1 suspicious for medullary thyroid carcinoma. Among ultrasonographic findings (Table 3), the significant predictors of malignant outcome were: hypoechoic or isoechoic patterns, peripheral vascularity, irregular margins, and calcification (any type). Although all other variables failed to reach statistical significance, comet‐tail artifact was only seen in benign cases, and spongiform features were more likely present in benign cases too.

**TABLE 2:** Univariate analysis of clinical variables. For each variable, the corresponding value that best contributed to differentiating the two groups is shown in parenthesis. “Hypothyroidism” and “hyperthyroidism” refer to the time of initial disease presentation. “Respiratory symptoms” refers to obstructive symptoms, including cough and shortness of breath

Clinical Variable	Final Diagnosis: Benign	Final Diagnosis: Malignant	*p* Value
Age	Median: 54 +/‐ 14	Mean: 47 +/‐ 16	0.15
Sex (female)	87 (92%)	29 (78%)	0.07
Previous Biopsy (Yes)	35 (38%)	8 (22%)	0.09
Palpable Nodule/Mass (Yes)	43 (45%)	24 (65%)	0.05
Hypothyroidism (Yes)	11 (12%)	0 (0%)	0.03
Hyperthyroidism (Yes)	8 (8%)	4 (11%)	0.74
Hoarseness (Yes)	14 (15%)	5 (14%)	1
Enlarging Mass (Yes)	21 (22%)	10 (27%)	0.65
Goiter (Yes)	49 (52%)	15 (41%)	0.33
Respiratory Symptoms (Yes)	15 (16%)	6 (16%)	1
Difficulty Swallowing (Yes)	35 (37%)	6 (16%)	0.02
Weight loss (Yes)	4 (4.2%)	3 (8.1%)	0.4
Radiation Exposure (Yes)	1 (1.1%)	1 (2.7%)	0.48
Familial history of thyroid cancer (Yes)	4 (4.2%)	0 (0%)	0.58
Familial history of thyroid disease (Yes)	17 (18%)	8 (22%)	0.63
Personal History of Thyroid Cancer (Yes)	1 (1.1%)	0 (0%)	1
Thyroid Medication (Yes)	9 (9.5%)	0 (0%)	0.06
Radioactive Iodine (Yes)	1 (1.1%)	3 (8.1%)	0.07

**TABLE 3:** Univariate analysis of ultrasonographic variables. For each variable, the corresponding value(s) that best contributed to differentiating the two groups is shown in parenthesis

Ultrasonographic Variable	Final Diagnosis: Benign	Final Diagnosis: Malignant	*p* Value
Nodule Size	Mean: 3.6 cm (SD: 2.2)	Mean: 3.8 cm (SD: 1.9)	0.47
Change in size (Yes)	8 (8.4%)	8 (50%)	0.07
Cystic (Yes)	17 (18%)	6 (16%)	1
Solid (Yes)	35 (37%)	15 (41%)	0.69
Complexity (Complex)	32 (34%)	12 (32%)	1
Spongiform (Yes)	10 (11%)	1 (2.7%)	0.18
Calcification (Yes)	15 (16%)	13 (35%)	0.02
Hypervascularity (Peripheral)	1 (1.1%)	7 (19%)	0.001
Echogenicity (Hypoechoic or Isoechoic)	17 (18%)	18 (50%)	0.001
Cervical Lymphadenopathy (Yes)	4 (4.2%)	2 (5.4%)	0.67
Margins (Irregular)	8 (8.4%)	8 (22%)	0.01
Comet Tail Artifact (Yes)	2 (2.1%)	0 (0%)	1
Fused Nodules (Yes)	4 (4.2%)	1 (2.7%)	1

For the multivariate analysis, 110 patients were assigned to the training group and 20 to the validation group. The final model included history of radioactive iodine administration, difficulty swallowing, previous thyroid biopsy, hypothyroidism, echogenicity, hypervascularity, margins, calcification, and cytology diagnosis as input parameters. The binary model was highly successful in determining the outcome (*p* value: 0.001) with an R^2^(Nagelkerke) score of 0.93. The area under the curve (AUC) as determined by receiver operating characteristics was 0.91. The overall accuracy of the model on the training dataset was 93% with a sensitivity of 92% and specificity of 96%, and, on the validation dataset, the accuracy was 80% with a sensitivity of 91% and specificity of 67%.

## 4 DISCUSSION

Management of clinically indeterminate thyroid nodules can be difficult due to their risk of malignancy, yet the majority are histologically benign.^6^ As a result, management can be highly variable and is necessarily dependent on factors beyond the cytology result such as patients’ health status, clinical and radiologic context, preferences of patients and/or clinicians, and other considerations. For example, management of AUS can range from clinical follow‐up with/without repeat FNA to surgical intervention. Similar variabilities are encountered in the management of SFN, where the extent of surgery may be more of a debate.^3^

Molecular testing has been increasingly utilized to assist with indeterminates as approximately seventy percent of differentiated thyroid cancers have detectable abnormalities involving BRAF, RAS, RET/PTC, or PAX8/PPAR gamma. However, because many of these may also be seen in benign neoplasms and in some hyperplastic nodules, interpretation of test results can be challenging. These tests usually follow one of the two approaches: 1. A “rule‐in” approach with a high positive predictive value that looks for the highly specific *BRAF V600E* mutation along with a mutational/translocation panel to increase sensitivity; 2. A “rule‐out” approach with high negative predictive power that assesses a comprehensive genetic expression profile which is used to identify very low risk nodules.^9^, ^12^, ^13^ While such tests may be beneficial in directing the management of some patients, they are clearly not stand alone tests, and their utilization is further limited by issues such as proper acquisition and handling of the specimen, accessibility, and insurance and monetary considerations both for the patients and health care system.^3^, ^14^

In fact, the interpretation of any diagnostic test should not be attempted independently of pre‐test probability; in other words, the post‐test probability is a product of pre‐test probability and the likelihood ratio attributed to the test. This is reflected in changes in positive and negative predictive values of tests due to baseline conditions including disease prevalence, sex, etc. For example, considering a test with 99% sensitivity and specificity, a patient with a pre‐test probability of 1% who tests positive would only have a post‐test probability of 40%.^15^ Thus, in this study, we endeavored to quantify the pre‐test probability of malignancy in patients undergoing thyroid FNA, based on clinical and ultrasonographic findings, and to use this in conjunction with their cytology results to calculate the post‐test probability of malignancy.

Our initial analysis confirmed, as expected, that benign, suspicious, and malignant FNA diagnoses are all reliable, independent predictors of surgical outcome and AUS and SFN are not (Table 1). Further, the rate of malignancy following indeterminate cytologic diagnoses in this study (AUS = 23%; SFN = 38%) is, although a bit on the high side, similar to that seen in the literature and supports the need for improved decision‐making tools to optimize the triage of patients.^6^ Our logistic regression model is such a tool, demonstrating a considerable improvement over cytology alone with an AUC of 0.91 and an overall accuracy of 93%.

**FIGURE 2:**
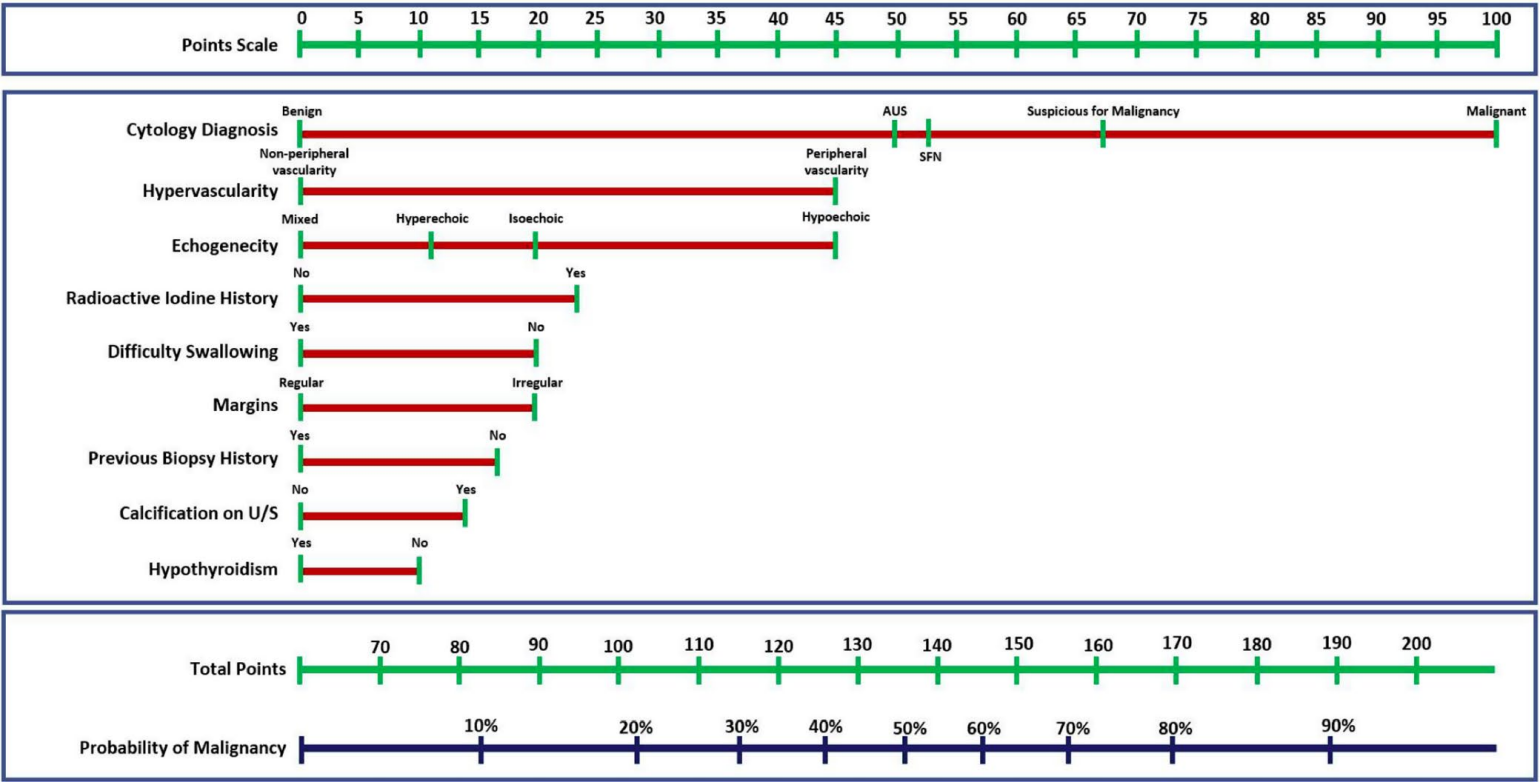
Nomogram for predicting the outcome of thyroidectomy based on cytologic, clinical, and ultrasonographic variables. Total point score determines the probability of malignancy

**FIGURE 3:**
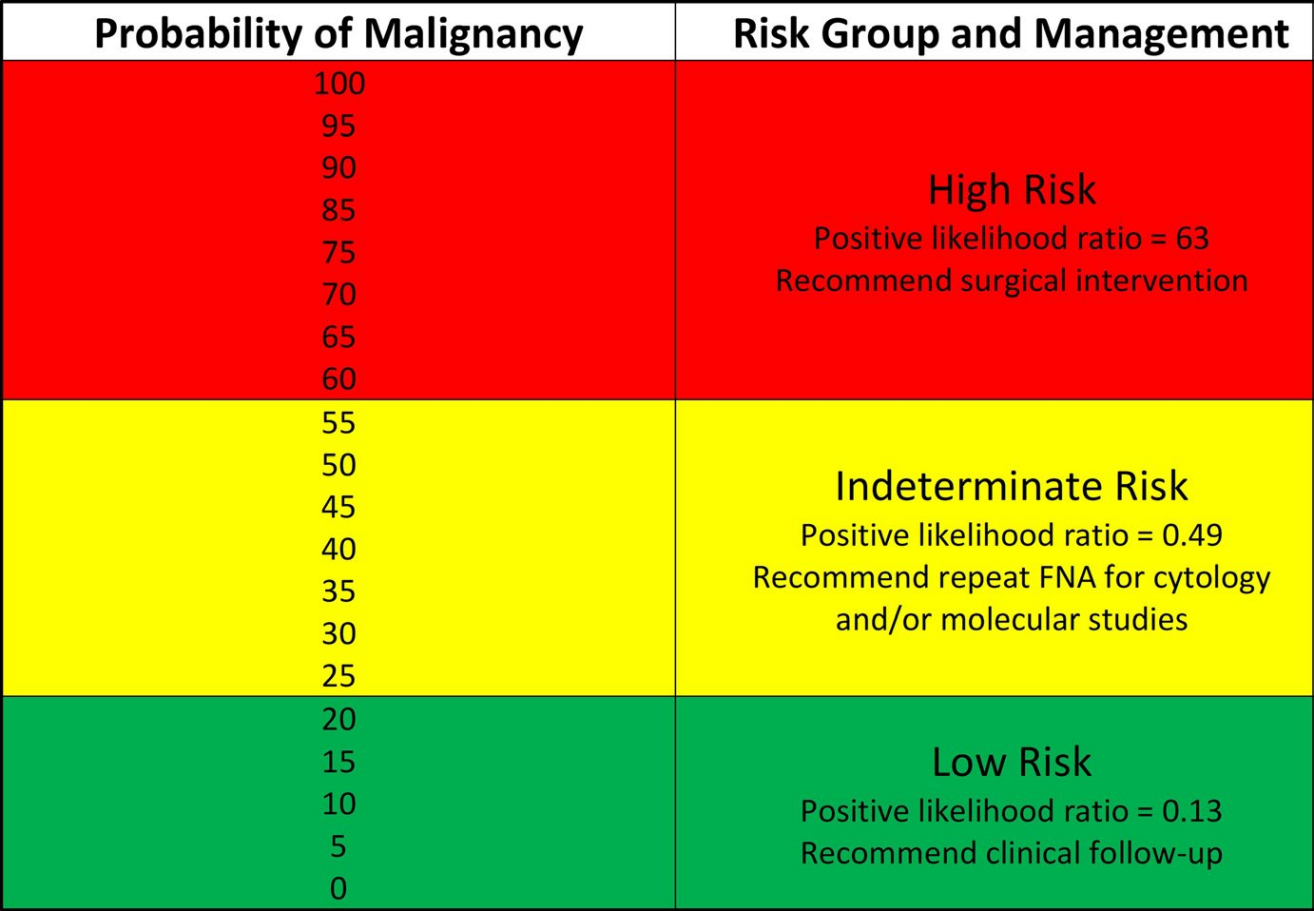
Nomogram derived risk stratification of the entire cohort of 130. Three probability (risk) groups with differing management plans can be established with the cutoffs shown

**FIGURE 4:**
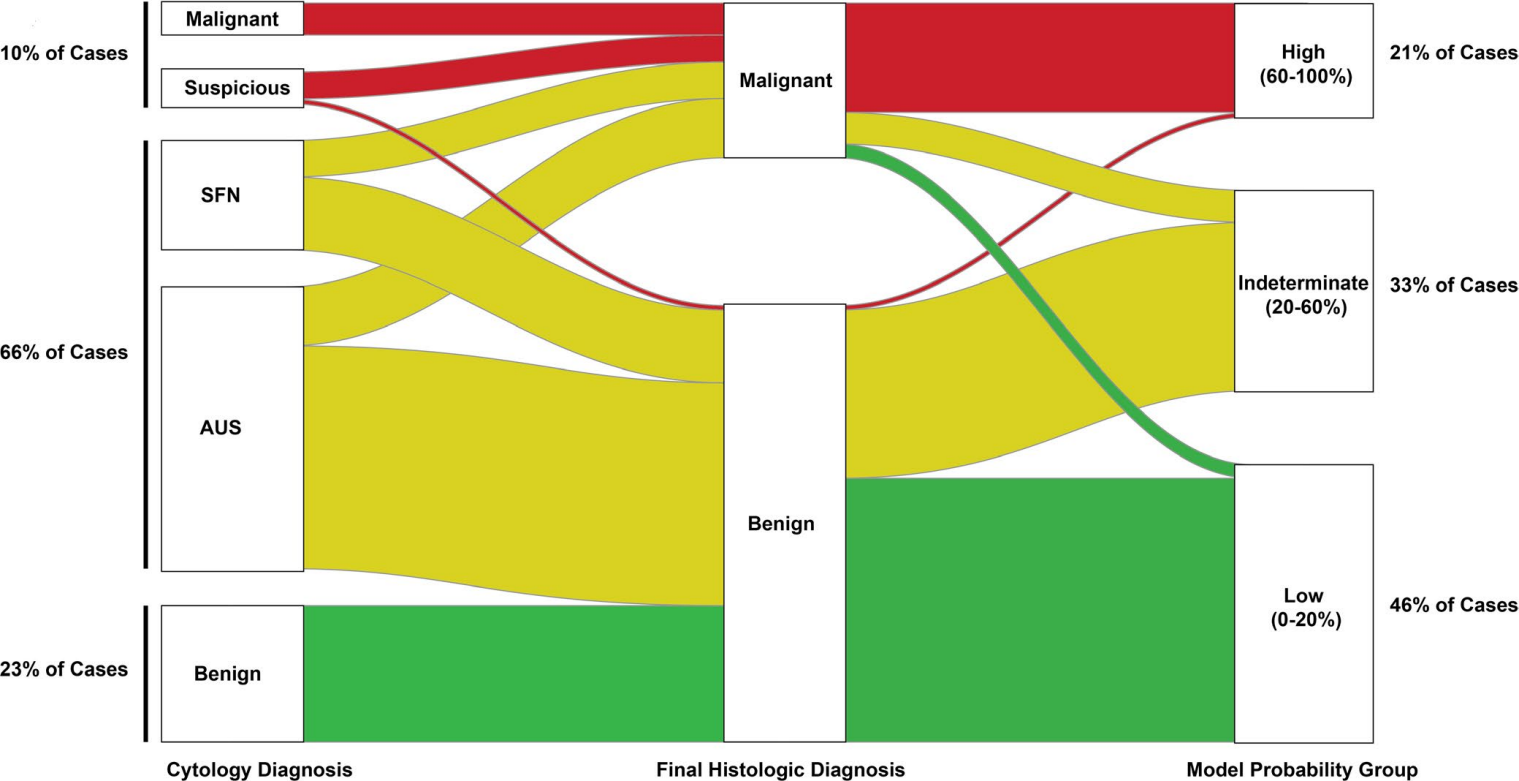
Alluvial graph showing the distribution of the 130 cases by outcomes and cytology diagnosis versus probability (risk) group with indeterminates in yellow. The curves connecting the columns on the left and right to the middle column show the proportion of each cytology or risk group that was benign or malignant

Additionally, we designed a nomogram to demonstrate the model's clinical application wherein scores for the individual variables are added up and the total score is used to calculate the post‐test probability of malignancy (Figure 2). Probability (risk) groups with distinct management recommendations can then be defined. Using the entire cohort of 130 to illustrate, a probability greater than 60% would correspond to a positive likelihood ratio of 63 for malignant outcome compared to a positive likelihood ratio of 0.13 when the probability is 20% or less (Figure 3, red and green, respectively). Patients with intermediate probabilities (i.e., a new indeterminate risk group) have more uncertain outcomes and these patients may be good candidates for additional testing (i.e., repeat FNA for cytology and/or molecular studies) versus diagnostic lobectomy. Using such cutoffs, only 33% of our cohort would be classified as indeterminate, whereas that number is double when considering indeterminate status by Bethesda category alone (Figure 4, yellow). The binary model, however, did misclassify four validation dataset cases: 1 was a follicular adenoma (with SFN cytology), 2 were nodular hyperplasia (both AUS), and the last was the cribriform‐morular variant of papillary thyroid carcinoma (also AUS). That said, all four cases fall into the indeterminate probability risk group for which further, conservative management would be recommended.

Although extensive attention has been paid to defining the risk of thyroid malignancy due to specific clinical, radiologic, or cytologic findings, relatively few studies have attempted to integrate this data.^16^, ^17^, ^18^, ^19^, ^20^, ^21^, ^22^ Angell et al. used a combination of histology and cytology outcomes where they considered all benign cytology results equivalent to a benign histology result. This powerful work risk stratifies based on demographics and ultrasound variables and includes a web‐based tool for estimating nodule risk. It does not include other relevant clinical variables, nor does it include cytology as an input variable. Hong et al. proposed combining the Korean College of Radiology's Thyroid Imaging Reporting and Data System (K‐TIRADS) scores with FNA results, but they did not account for any clinical information.^17^ Their cytology‐ultrasonography (CU) scoring system consisted of four groups analogous to Bethesda categories. However, this CU system had a considerable number of indeterminates (CU categories II and III) with widely variable risks of malignancy. In addition to including clinical data, we opted out of incorporating any specific sonographic scoring system due, in part, to the lack of any being universally accepted and reported at present.

Ianni et al. proposed a clinical, ultrasonographic, and FNA scoring system where cytologic diagnoses were made in accordance with the five categories of the Italian Working Group SIAPEC‐IAP (Societa` Italiana di Anatomia Pathologica e Citopatologia Diagnostica‐International Academy of Pathology), a system which is similar to and slightly predates Bethesda.^23^ Ianni's group developed a cancer risk score for the preoperative assessment of thyroid nodules, the “CUT” score.^18^ Although this study had a similar approach to ours, the CUT score is more difficult to interpret as it employs a clinical plus ultrasonographic score (C + U) and a separate cytology score (T). Additionally, they considered only a limited number of clinical variables, instead, placing a greater emphasis on ultrasonography, and their cytology score is not directly translatable to The Bethesda System. Despite these differences, the overall predictive ability of their model is comparable to ours with a reported AUC of 0.904, and this further validates the use of a multivariate approach to calculate post‐test probabilities and guide the management of nodules after FNA.

We are not the first to suggest the use of a nomogram in the management of thyroid nodules. Nixon et al. is a comparable study of similar size that demonstrated high accuracy, but it also predated The Bethesda System.^20^ They instead assessed individual microscopic variables (e.g., nuclear grooves, presence of colloid) rather than categorical diagnoses, and their work is, therefore, not translatable to current clinical practice. Yoon's group presented a nomogram based on a large number of purely Bethesda III cases and they correlated only ultrasound with outcomes.^21^ Thus, it is not surprising that their more limited model is less accurate (AUC 0.817). More recently, Öcal et al. published a large study that included all Bethesda categories as we did.^22^ However, their reported rate of malignancy following AUS is exceptionally high (44%), which suggests significant overutilization of this category, and this may be one explanation for their lower AUC of 0.784.

Although there is a potential bias in our cohort due to its restriction to surgically managed nodules, it is challenging to better define outcome; of note, some related studies have evaluated clinical and sonographic variables against cytologic (rather than histologic) outcome, but this approach is suboptimal and prohibits the use of cytology as an input variable.^16^, ^24^, ^25^ Alternately, while long‐term clinical follow‐up and/or “benign” FNA molecular testing might be utilized as outcome surrogates for non‐surgically managed nodules, they each have significant limitations, notably, the variable accuracy of the different available molecular tests and the slow progression of most thyroid cancers. Moreover, only one of our three institutions utilized any molecular testing during the study period, and it did so uncommonly and inconsistently.

The lack of standardized reporting for diagnostic sonography in the medical record both among and within the three institutions should also be highlighted. For example, reports may have favored highlighting concerning sonographic features over more benign ones, and, thus, comet‐tail artifact and spongiform appearance may have failed to reach significance due to underreporting rather than to a lack of predictive ability. Similarly, perhaps peripheral rather than central vascularity is associated with malignancy because of inconsistent reporting or interpretation of vascularity patterns. However, the significance of vascularity as a risk factor for malignancy is controversial.^26^ In our cohort, from the 16 cases that reported central vascularity, 8 had a final histologic diagnosis of thyroid carcinoma, and 8 had a benign final histologic diagnosis. Additionally, it would have been interesting to evaluate ultrasound scoring systems’ (e.g., TI‐RADS’s) ability to predict outcomes. This could not be attempted, however, due to the frequent absence of this information. Diagnostic sonography reports instead more commonly recommended biopsy (or not) of specific, described nodules rather than specifying a TI‐RADS or other system's risk category of the nodules.^27^

Our model's apparent utility in reducing indeterminate nodules is exciting and the next step is validation in an independent test population. Validation is also prudent due to the high prevalence of malignancy among indeterminate Bethesda diagnoses in our data set, 61% versus an expected 45% or less.^6^ This is not surprising though in the typical practice setting where cytology is, at least in part, read by pathologists who are not fellowship‐trained and board certified. It is our experience that such pathologists tend to diagnose more conservatively and, when reviewing their cases in consultation, many Bethesda III–V diagnoses are reclassified to a higher Bethesda category. In Olson et al., a second cytopathology review at a tertiary center resulted in the reclassification of 1238 thyroid FNA cases (32%), and, among the reclassified cases in the subset of 1049 with histologic follow up, the appropriate upgrades in Bethesda category to malignancy were significantly greater than the inappropriate downgrades away from malignancy (*p* value: 0.001).^28^ It is for this reason that prospective intradepartmental slide consultation with a boarded cytopathologist for any Bethesda impression greater than II is desirable for diagnostic quality assurance, and, in our study cohort, it appears that indeterminate Bethesda diagnoses may be overrepresented due to such an “underdiagnosis” phenomenon. That said, the absence of false‐negative cytologic diagnoses in our data set is reassuring. We did consider incorporating both retrospective cytology slide and sonography image reviews in our design, but implementation proved challenging. Moreover, because both fields are well known to have subjective and experiential components, it is reasonable that any predictive model be subject to these.^28^, ^29^

Our choice to place the two NIFTPs in the benign outcome group may be controversial. It could be argued that, because surgery is presently the standard of care for these low‐risk neoplasms, it would be more appropriate to place them in the malignant outcome group. However, we assigned the outcome by biologic behavior (i.e., whether or not malignant behavior would be expected) rather than by whether or not surgical management was appropriate, and both NIFTPs are classified as indeterminate by our model. For comparison, we also re‐ran the analysis with NIFTP alternately regarded as a “malignant” outcome, and the changes in the model's parameters and metrics were negligible with both NIFTPs again regarded as indeterminate. In contrast to our model, preoperative cytologic classification of proven NIFTPs is known to span Bethesda categories, and false‐positive cytologic classifications are a concern due to unintended psychological and clinical consequences.^11^, ^12^ Thus, the classification of NIFTP as indeterminate by our model seems optimal and should allow for correct, conservative management—either additional testing (with molecular studies expected to move at least 90% of NIFTPs to lobectomy) versus lobectomy.^30^ Nevertheless, subsequent larger studies will undoubtedly be of value in assessing how best to group NIFTPs, and any predictive model must be assessed for its ability to appropriately direct care for these unusual neoplasms.

We report a thorough and clinically applicable multivariate analysis of predictors of thyroid nodule malignancy encompassing clinical, radiologic, and cytologic variables. We demonstrate the use of a nomogram as a sensitive tool that can simplify communication to patients of their individualized malignancy risk. In this way, evidence‐based management plans can also be clearly defined allowing for triage to surgery, additional testing for indeterminates, or clinical/sonographic follow‐up. Most importantly, this approach may translate into a significant reduction of indeterminate nodules (i.e., the gray zone) as compared to relying alone on cytology for risk‐stratification, which is highly desirable due to the limitations of ancillary molecular testing and the need to mitigate surgical overtreatment. Confirmation of these findings in an independent surgical cohort is the next step, which may also serve to refine and/or alter the significance of some variables in the nomogram. If the model performs well there, a randomized controlled trial would be the final step before implementation into practice.

## CONFLICTS OF INTEREST

The authors have no conflicts of interest to report.

## ETHICAL APPROVAL STATEMENT

Ethical approval is waived. This study was determined to be exempt from review by the IRB at LSU Health Sciences Center, New Orleans.

## Data Availability

The datasets generated during and analyzed during the current study are available from the corresponding author on reasonable request.
